# Protective Effect of Curcumin on Pulmonary and Cardiovascular Effects Induced by Repeated Exposure to Diesel Exhaust Particles in Mice

**DOI:** 10.1371/journal.pone.0039554

**Published:** 2012-06-22

**Authors:** Abderrahim Nemmar, Deepa Subramaniyan, Badreldin H. Ali

**Affiliations:** 1 Department of Physiology, Faculty of Medicine and Health Sciences, United Arab Emirates University, Al Ain, United Arab Emirates; 2 Department of Pharmacology and Clinical Pharmacy, College of Medicine & Health Sciences, Sultan Qaboos University, Al-Khod, Sultanate of Oman; University of Pittsburgh, United States of America

## Abstract

Particulate air pollution has been associated with increased risk of cardiopulmonary diseases. However, the underlying mechanisms are not fully understood. We have previously demonstrated that single dose exposure to diesel exhaust particle (DEP) causes lung inflammation and peripheral thrombotic events. Here, we exposed mice with repeated doses of DEP (15µg/animal) every 2^nd^ day for 6 days (a total of 4 exposures), and measured several cardiopulmonary endpoints 48 h after the end of the treatments. Moreover, the potential protective effect of curcumin (the yellow pigment isolated from turmeric) on DEP-induced cardiopulmonary toxicity was assessed. DEP exposure increased macrophage and neutrophil numbers, tumor necrosis factor α (TNF α) in the bronchoalveolar lavage (BAL) fluid, and enhanced airway resistance to methacoline measured invasively using Flexivent. DEP also significantly increased plasma C-reactive protein (CRP) and TNF α concentrations, systolic blood pressure (SBP) as well as the pial arteriolar thrombosis. It also significantly enhanced the plasma D-dimer and plasminogen activator inhibitor-1 (PAI-1). Pretreatment with curcumin by oral gavage (45 mg/kg) 1h before exposure to DEP significantly prevented the influx of inflammatory cells and the increase of TNF α in BAL, and the increased airway resistance caused by DEP. Likewise, curcumin prevented the increase of SBP, CRP, TNF α, D-dimer and PAI-1. The thrombosis was partially but significantly mitigated. In conclusion, repeated exposure to DEP induced lung and systemic inflammation characterized by TNFα release, increased SBP, and accelerated coagulation. Our findings indicate that curcumin is a potent anti-inflammatory agent that prevents the release of TNFα and protects against the pulmonary and cardiovascular effects of DEP.

## Introduction

A number of epidemiological studies reported strong and consistent associations between exposure particulate air pollution and increase of respiratory and cardiovascular morbidity and mortality [1], [2]. In this context, epidemiological time-series studies have identified an association between daily changes in concentration of ambient air pollution and daily number of deaths and hospitalizations, particularly from cardiovascular disease and following relatively short time lags after exposure peaks [1], [2]. It has been suggested that traffic-derived particles, of which diesel exhaust particles (DEP) are major contributor, are the most toxic component [1], [2]. Moreover, the ambient level of black carbon particles, used as a tracer for traffic pollution, has been consistently associated with a variety of adverse health outcomes [1], [2].

A number of possible mechanisms have been suggested to explain these effects, including direct effects of particles that translocated into the systemic circulation, disturbances of the cardiac autonomic nervous system, and pulmonary and systemic oxidative stress and inflammatory responses that trigger endothelial dysfunction, atherosclerosis, and coagulation [1], [3]. However, the exact mechanistic pathways are still not fully understood.

Human studies have previously demonstrated that controlled exposure to DEP results in endothelial dysfunction, impaired endogenous fibrinolysis, and increased thrombus formation in both healthy human subjects and in patients with stable coronary heart disease [4], [5]. Similarly, DEP impairs endothelium-dependent vasodilatation in animal studies both *in vivo* and *ex vivo* [2]. We have recently demonstrated that single dose pulmonary exposure to DEP (up to 24 h) induces pulmonary and systemic inflammation and the occurrence of thrombotic events in the femoral vein and artery of hamsters and cerebral microvessels of mice [6]–[8]. However, the effect repeated exposure of DEP on airway inflammation and resistance and pial arteriole thrombosis and markers of coagulation has not been reported so far.

Curcumin is the major yellow pigment in turmeric (the ground rhizome of *Curcuma longa* Linn), which is widely used as a spice and coloring agent in several foods, as well as cosmetics and drugs [9], [10]. Recently, curcumin has been identified as an inhibitor of oxidant-, cytokine-, and cigarette smoke-induced NF-_κ_B activation in human lung epithelial cell lines [11]. Indeed, oral curcumin administration has been reported to inhibit bleomycin-induced pulmonary fibrosis in rats [12] and cigarette smoke-induced lung inflammation and emphysema in mice [13]. However, to our knowledge no study, to date, has addressed the effect of curcumin on the pulmonary and cardiovascular effects of DEP.

Consequently, in the present study, we have assessed the effect of repeated exposure to DEP (15 µg/animal) 48 h after the last of four exposures to DEP performed every second day on a comprehensive set of indices of respiratory endpoints including pulmonary inflammation and airway resistance measured invasively using forced oscillation as well as cardiovascular parameters, including blood pressure, pial arterioles thrombosis and markers of inflammation and fibrinolysis. Moreover, we assessed the possible protective effect of curcumin on DEP-induced pulmonary and cardiovascular events.

## Materials and Methods

### Ethics Statement

This project was reviewed and approved by the Institutional Review Board of the United Arab Emirates University, Faculty of Medicine and Health Sciences, and experiments were performed in accordance with protocols approved by the Institutional Animal Care and Research Advisory Committee.

### Particles

Diesel exhaust particles (DEP; SRM 2975), obtained from the National Institute of Standards and Technology (NIST, Gaithersburg, MD, USA), were suspended in sterile normal saline (NaCl 0.9 %) containing Tween 80 (0.01 %). To minimize aggregation, particle suspensions were always sonicated (Clifton Ultrasonic Bath, Clifton, New Jersey, USA) for 15 min and vortexed before their dilution and prior to intratracheal (i.t.) administration. Control animals received normal saline containing Tween 80 (0.01 %). We have previously [14] analyzed the size of DEP used in the present study by transmission electron microscopy, and found a substantial amount of ultrafine (nano) sized of carbonaceous particle aggregates and larger particle aggregates (< 1 µm in largest diameter). Geometric mean aerodynamic diameter of 215°nm generated from the same DEP material have been previously reported [15].

### Animals and treatments

Male TO mice (30–35 g, HsdOla∶TO, Harlan, UK) were housed in light (12-h light∶12-h dark cycle) and temperature-controlled (22±1°C) rooms. They had free access to commercial laboratory chow and were provided tap water *ad libitum*.

**Figure 1:**
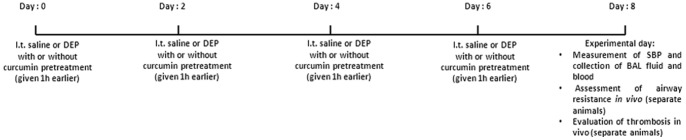
Treatments and endpoints following the repeated intratracheal instillation (i.t.) of saline or diesel exhaust particles (DEP) with or without curcumin pretreatment (given by oral gavage) in mice. SBP: systolic blood pressure; BAL: bronchoalveolar lavage.

Mice were anesthetized with sodium pentobarbital (60 mg/kg, i.p.), placed supine with extended neck on an angled board. A Becton Dickinson 24 Gauge cannula was inserted via the mouth into the trachea. Either the DEP suspensions (15 µg/mouse) or saline-only were instilled intratrachealy (i.t.) (50 µl) via a sterile syringe and followed by an air bolus of 50 µl every other day. DEP (15 µg/mouse) or saline were i.t. administered on days 0, 2, 4, 6, and 48 hours after the last exposure to DEP, various pulmonary and cardiovascular endpoints were measured (figure 1). Eight mice were included in each group.

### Systolic blood pressure (SBP) measurement

48 hours after the last exposure to DEP, the systolic BP (SBP) was measured using a computerized noninvasive tail-cuff manometry system (ADInstrument, Colorado Springs, USA). To avoid procedure-induced anxiety, mice were trained for 5 consecutive days before the experimental procedure [16].

### Blood collection and analysis of bronchoalveolar lavage (BAL) fluid

48 hours after the last i.t. administration of either saline or DEP, the animals were anesthetized, as described above, and blood was drawn from the inferior vena cava in EDTA (4 %). A sample was used for platelets and white blood cells (WBC) counts using an ABX VET ABC Hematology Analyzer with a mouse card (ABX Diagnostics, Montpellier, France). The remaining blood was centrifuged at 4°C for 15 min at 900 *g* and the plasma samples were stored at –80°C until further analysis.

Mice were then killed with an overdose of sodium pentobarbital. The trachea was cannulated and lungs were lavaged three times with 0.7 ml (a total volume of 2.1 ml) of sterile NaCl 0.9 %. The recovered fluid aliquots were pooled. No difference in the volume of collected fluid was observed between the different groups. BAL fluid was centrifuged (1,000 *g* × 10 min, 4°C). Cells were counted after resuspension of the pellets and the differentials were microscopically performed on cytocentrifuge preparations fixed in methanol and stained with Diff Quick (Dade, Brussels, Belgium). The supernatant was stored at −80 °C until further analysis.

In the BAL fluid, the concentrations of tumor necrosis factor α and IL-6 were determined using ELISA Kits (R & D systems, Minneapolis, MN).

### Airway reactivity to methacholine

In separate animals, airway hyperreactivity responses were measured using a forced oscillation technique (FlexiVent, SCIREQ, Montreal, Canada). Airway resistance (R) was assessed after increasing exposures to methacholine. Mice were anesthetized with an intraperitoneal injection of pentobarbital (70 mg/kg). The trachea was exposed, and into it, an 18-gauge metal needle was inserted. Mice were connected to a computer-controlled small animal ventilator and quasi-sinusoidally ventilated with a tidal volume of 10 ml/kg at a frequency of 150 breaths/min and a positive end-expiratory pressure of 2 cm H_2_O to achieve a mean lung volume close to that during spontaneous breathing. After measurement of a baseline, each mouse was challenged with methacholine aerosol, generated with an in-line nebulizer and administered directly through the ventilator for 5s, with increasing concentrations (0, 0.625, 1.25, 2.5, 5, and 10 mg/ml). Airway resistance (R) was measured using a "snapshot" protocol each 20 s for 2 min. The mean of these six values was used for each methacholine concentration, unless the coefficient of determination of a measurement was smaller than 0.95. For each mouse, R was plotted against methacholine concentration (from 0 to 10 mg/ml) [17].

### Determination of IL-6, TNFα, C-reactive protein (CRP), D-dimer and plasminogen activator inhibitor-1 (PAI-1) concentrations in plasma

The concentrations of mouse IL-6, TNF α (R & D systems, Minneapolis, MN, USA), PAI-1 (Molecular Innovation, Southfield, MI, USA), D-dimer (Uscn Life Science Inc, Wuhan, China) and CRP (Uscn Life Science Inc, Wuhan, China) were determined using ELISA Kits.

### Experimental pial cerebral arterioles thrombosis model

In a separate experiment, *in vivo* pial arterioles thrombogenesis was assessed 48 hours after the last i.t. instillation of either DEP or saline, according to a previously described technique [7], [8]. Briefly, the trachea was intubated after induction of anesthesia with urethane (1 mg/g body weight, i.p.), and a 2F venous catheter (Portex, Hythe, UK) was inserted in the right jugular vein for the administration of fluorescein (Sigma, St. Louis, MO, USA). After that, a craniotomy was first performed on the left side, using a microdrill, and the dura was stripped open. Only untraumatized preparations were used, and those showing trauma to either microvessels or underlying brain tissue were discarded. The animals were then placed on the stage of a fluorescence microscope (Olympus, Melville, NY, USA) attached to a camera and DVD recorder. A heating mat was placed under the mice and body temperature was raised to 37°C, as monitored by a rectal thermoprobe connected to a temperature reader (Physitemp Instruments, NJ, USA). The cranial preparation was moistened continuously with artificial cerebrospinal fluid of the following composition (mM): NaCl 124, KCl 5, NaH_2_PO_4_ 3, CaCl_2_ 2.5, MgSO_4_ 4, NaHCO_3_ 23 and glucose 10, pH 7.3–7.4. A field containing arterioles 15–20 µm in diameter was chosen. Such a field was taped prior to and during the photochemical insult, which was carried out by injecting fluorescein (0.1 ml/mouse of 5 % solution) via the jugular vein, which was allowed to circulate for 30–40 sec. The cranial preparation was then exposed to stabilized mercury light. The combination produces endothelium injury of the arterioles. This, in turn, causes platelets to adhere at the site of endothelial damage and then aggregate. Platelet aggregates and thrombus formation grow in size until complete vascular occlusion. The time from the photochemical injury until full vascular occlusion (time to flow stop) in arterioles were measured in seconds. At the end of the experiments, the animals were euthanized by an overdose of urethane.

### Effect of curcumin pretreatment on pulmonary and systemic parameters

Curcumin (also named diferuloylmethane) [(E, E)-1,7-bis(4-hydroxy-3-methoxyphenyl)-1,6-heptadiene-3,5-dione, Sigma] 45 mg/kg [18] was suspended in 200 µl of vehicle (0.5 % methylcellulose; Sigma) or 200 µl of vehicle alone was administrated by oral gavage to mice [18] 1 h before each i.t. saline or DEP, and all the parameters described above were evaluated (figure 1). The dose of curcumin administered was chosen to approximate, on a weight per weight basis, curcumin doses that have been well tolerated in humans in previous studies [18].

### Statistics

Data were analyzed with GraphPad Prism Version 4.01 for Windows software (Graphpad Software Inc., San Diego, USA) and expressed as means ±SEM. Data were tested for normal distribution using the Kolmogorov–Smirnov test. After that, one-way analysis of variance (ANOVA), followed by Bonferroni *post-hoc* test, was used to determine differences between the different groups. P values less than 0.05 were considered significant.

## Results

### **Cell composition and number in BAL fluid**

**Figure 2:**
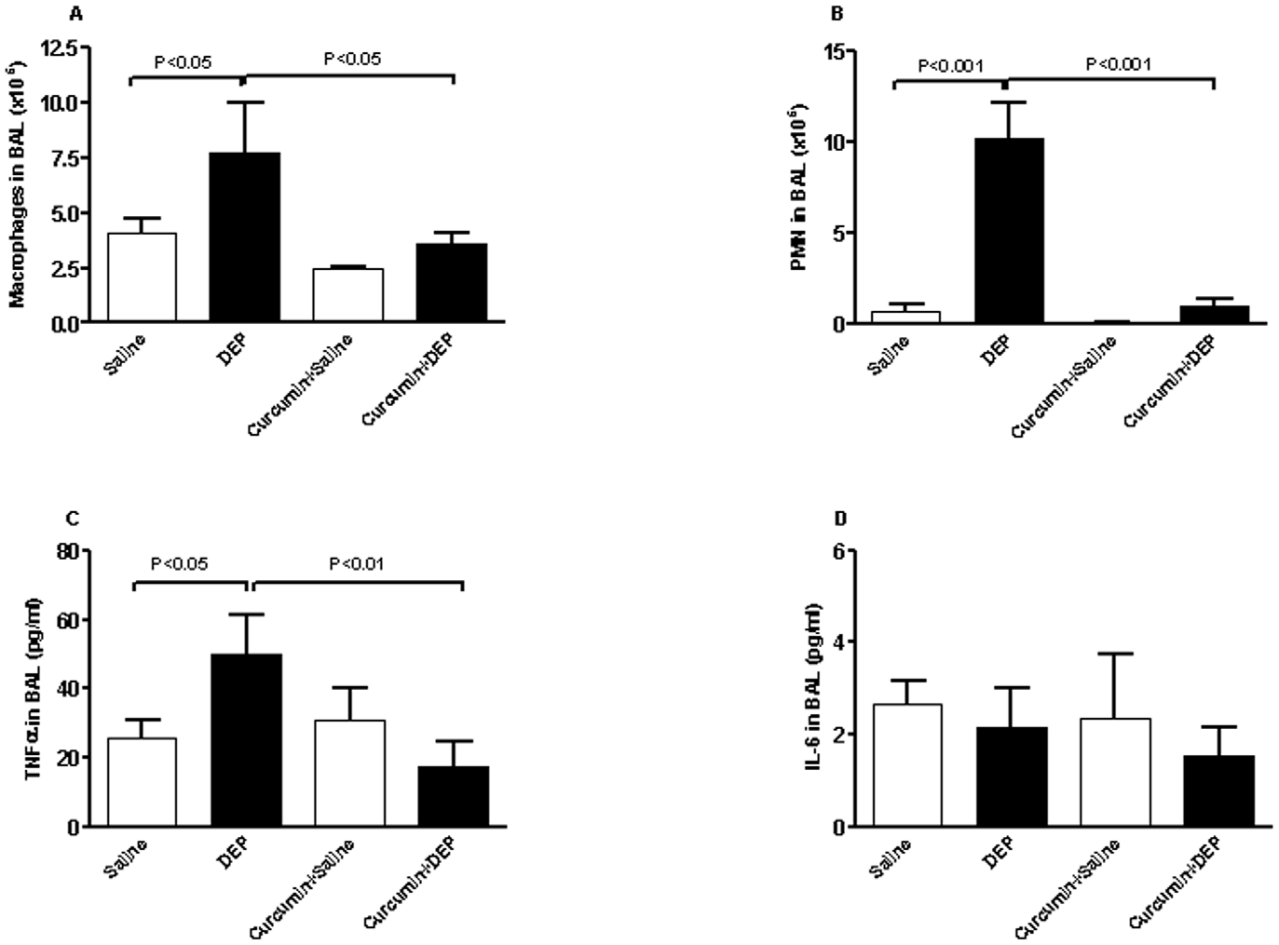
Number of macrophages (A) and polymorphonuclear neutrophils (PMN) (B), and tumor necrosis factor α (TNF α, C) and interleukin-6 (IL-6, D ) concentrations in bronchoalveolar lavage, after repeated intratracheal instillation of saline or diesel exhaust particles (DEP, 15 µg/animal) with or without curcumin pretreatment. Data are mean ± SEM (n = 8 in each group). Statistical analysis by one-way analysis of variance (ANOVA), followed by Bonferroni multiple-range tests.

Depending on the i.t. treatment performed, the cells found in BAL were primarily macrophages and PMN (figure 2A–B). Lymphocytes were not found in control mice BAL. No other cells were observed microscopically.

Repeated i.t. administration of DEP caused a marked and significant increase in macrophages. Similarly, repeated exposure to DEP caused a significant increase in PMN numbers. TNFα concentrations (but not IL-6) were also increased following the i.t. instillation of DEP in mice. Curcumin pretreatment alone did not affect the cell numbers or the measured cytokines. However, curcumin pretreatment significantly prevented the influx of inflammatory cells and the increase of TNFα concentrations in BAL.

### Airway hyper-reactivity to methacholine

**Figure 3:**
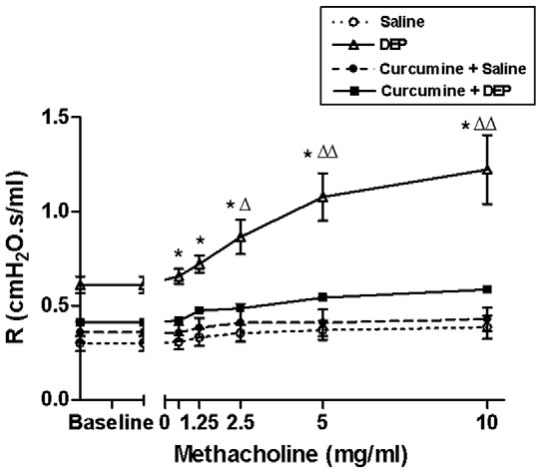
Airway hyper-responsiveness. The airway resistance (*R*), after increasing concentrations of methacholine (0–10 mg/ml), was measured via the forced oscillation technique (FlexiVent) after repeated intratracheal instillation of saline or diesel exhaust particles (DEP, 15 µg/animal) with or without curcumin pretreatment. Data are mean ±SEM (n = 8 in each group). Statistical analysis by one-way analysis of variance (ANOVA), followed by Bonferroni multiple-range tests. <$>\scale 80%\raster="rg1"<$> indicates P<0.001 between DEP and saline groups for the same methacholine concentration. Δ: indicates P<0.01 between DEP and curcumin+DEP groups for the same methacholine concentration. ΔΔ: indicates P<0.001 between DEP and curcumin+DEP groups for the same methacholine concentration.

Figure 3 shows airway resistance to increasing concentrations of methacholine after repeated exposure to either saline or DEP with or without curcumin pretreatment. Repeated exposure to DEP caused a significant and dose-dependent increase in the airway resistance compared with the saline-treated group. No statistical differences were observed between the groups treated with saline or curcumin+saline. Interestingly, curcumin pretreatment completely prevented DEP-induced enhancement of airway resistance after increasing concentrations of methacholine.

### CRP, TNF α and IL-6 concentrations in plasma

**Figure 4:**
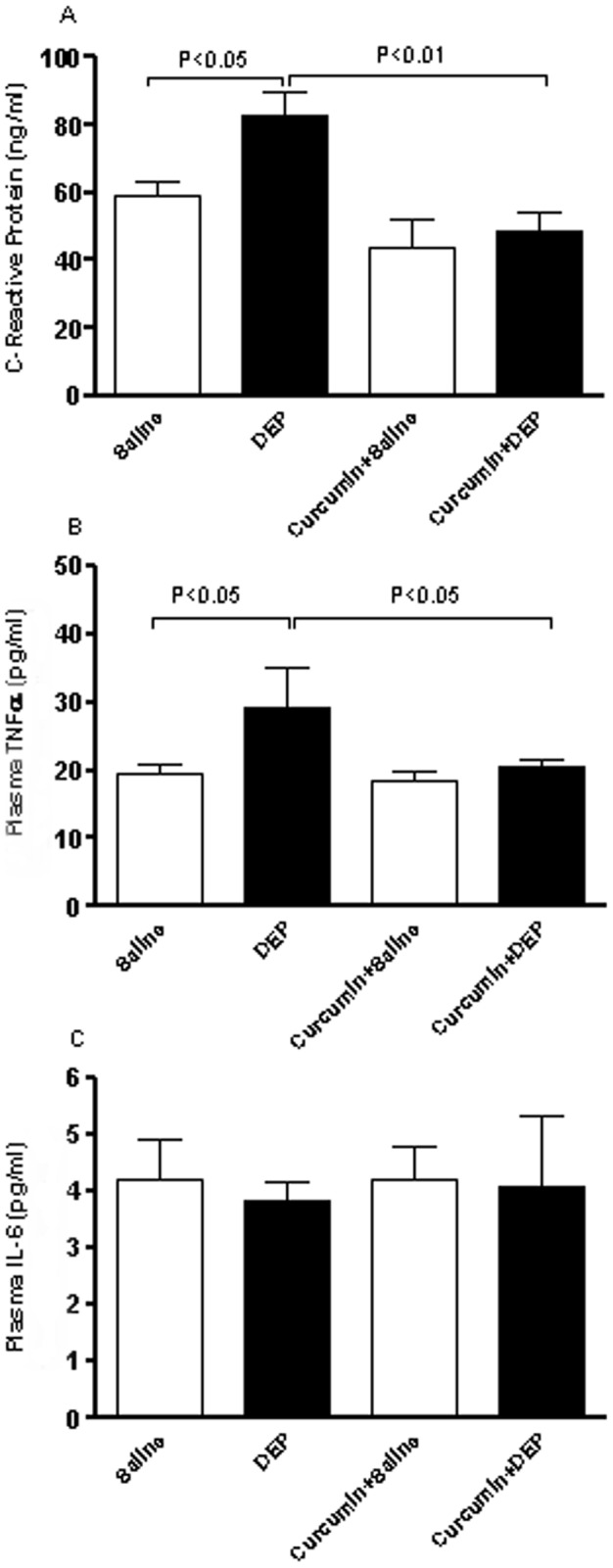
C-reactive protein (CRP, A), tumor necrosis factor α (TNF α, B) and interleukin-6 (IL-6, C) concentrations in plasma, after repeated intratracheal instillation of saline or diesel exhaust particles (DEP, 15 µg/animal) with or without curcumin pretreatment. Data are mean±SEM (n = 8 in each group). Statistical analysis by one-way analysis of variance (ANOVA), followed by Bonferroni multiple-range tests.

Repeated exposure to DEP caused systemic inflammation evidenced by a significant rise in CRP (figure 4A) and TNF α (figure 4B) (but not IL-6, figure 4C) concentrations in plasma compared with saline-treated group. Curcumin pretreatment alone did not significantly affect the measured inflammatory markers. Pretreatment of mice with curcumin significantly reversed the increases in CRP (figure 4A) and TNFα (figure 4B) caused by the repeated exposure to DEP.

### Systolic blood pressure (SBP)

**Figure 5:**
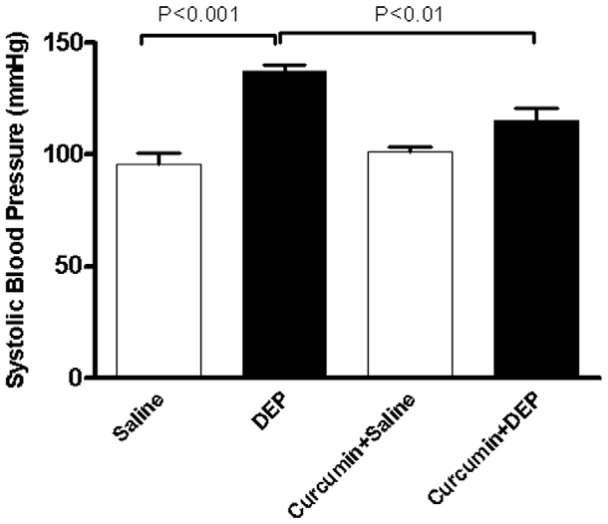
Systolic blood pressure, after repeated intratracheal instillation of saline or diesel exhaust particles (DEP, 15 µg/animal) with or without curcumin pretreatment. Data are mean±SEM (n = 8 in each group). Statistical analysis by one-way analysis of variance (ANOVA), followed by Bonferroni multiple-range tests.

Figure 5 illustrates the effect of repeated i.t. administration of DEP on SBP in mice. Compared to that in the controls, repeated exposure to DEP exhibited a significant increase in SBP. While treatment with curcumin+saline did not affect SBP, curcumin significantly prevented the increase of SBP induced by repeated i.t. instillation of DEP.

### Platelet numbers in blood and photochemically-induced thrombosis in pial arterioles

**Figure 6:**
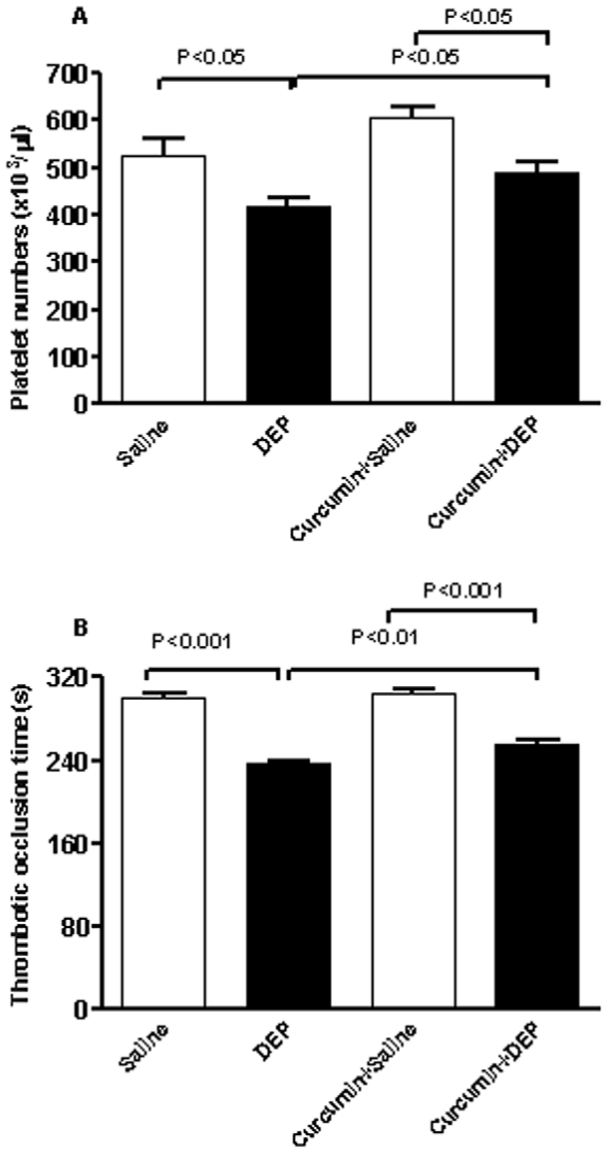
Circulating platelets numbers (A) and occlusion time in pial arterioles (B), after repeated intratracheal instillation of saline or diesel exhaust particles (DEP, 15 µg/animal) with or without curcumin pretreatment. Data are mean±SEM (n = 8 in each group). Statistical analysis by one-way analysis of variance (ANOVA), followed by Bonferroni multiple-range tests.

Platelet counts in blood were significantly decreased by repeated exposure to DEP compared with control mice (figure 6A), indicating the occurrence of platelet aggregation *in vivo*. Pretreatment with curcumin did not affect the circulating platelet numbers. However, curcumin pretreatment partially and significantly prevented the decrease in platelet numbers caused by repeated exposure to DEP (figure 6A).

In line with the results of platelet numbers, compared to control group, repeated exposure to DEP induced a shortening of the occlusion time in pial arterioles in a photochemically injured vessel. Curcumin alone did not affect the thrombotic occlusion time. In mice pretreated with curcumin, there was a partial and significant abrogation in DEP-induced shortening of the occlusion time in pial arterioles (Figure 6B).

### D-dimer, PAI-1 and vWF plasma concentrations

**Figure 7:**
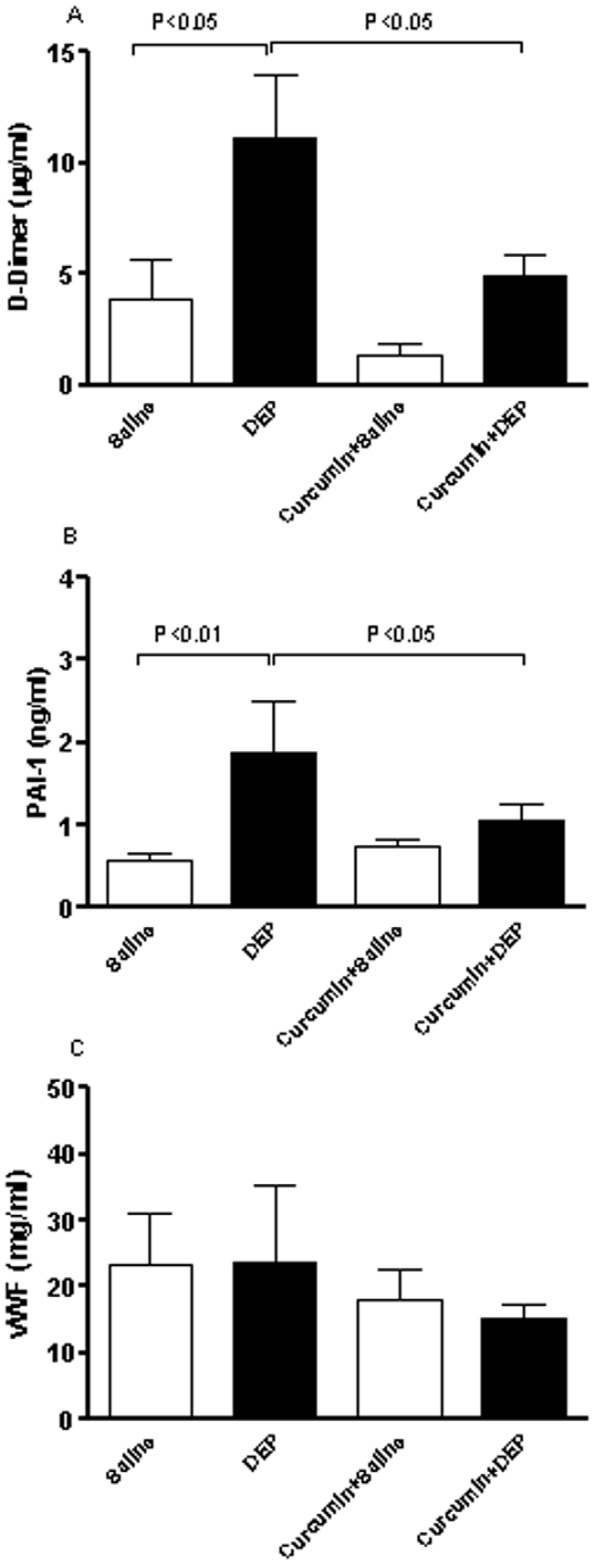
Plasma concentrations of D-dimer (A), plasminogen activator inhibitor 1 (PAI-1, B) and von Willebrand factor (vWF, C), after repeated intratracheal instillation of saline or diesel exhaust particles (DEP, 15 **µg/animal) with or without curcumin pretreatment.** Data are mean0±SEM (n = 8 in each group). Statistical analysis by one-way analysis of variance (ANOVA), followed by Bonferroni multiple-range tests.

Figure 7 illustrates the effect of repeated exposure to DEP on the plasma concentration of D-dimer (figure 7A), PAI-1 (figure 7B) and vWF (figure 7C). Compared to control group, repeated exposure to DEP caused a significant increase in D-dimer. Likewise the plasma concentrations of PAI-1 were significantly increased by the repeated i.t. instillation of DEP. In contrast, the concentrations of vWF in plasma were not significantly affected by DEP. Curcumin alone did not affect the plasma D-dimer concentration but it significantly prevented its increase caused by repeated exposure to DEP. Similarly, pretreatment with curcumin alone did not affect the plasma PAI-1 levels. However, curcumin significantly inhibited the increase of PAI-1 induced by DEP.

## Discussion

The present work provides experimental evidence that repeated exposure to DEP induces lung and systemic inflammation and airway hyperreactivity, increases SBP, and accelerates coagulation. TNF α production was increased both in BAL and plasma. Interestingly, pretreatment with curcumin significantly prevented the respiratory and cardiovascular effect and inhibited the release of TNF α.

In the present study, we assessed the effect of repeated exposure to DEP on respiratory and cardiovascular endpoints. This approach is more relevant to human exposure scenarios than single dose exposure. The dose used here is close to the range of PM_10_ to which humans might be exposed [19]. In 2002, the United States Environmental Protection Agency described a range of maximal city PM_10_ concentrations between 26 and 534 μg/m^3^ [20]. Numerous mega cities in the world have much greater levels of PM_10_, with annual averages of 200 to 600 μg/m^3^ and peak concentrations frequently exceeding 1,000 μg/m^3^ [21]. Using the highest value in the United States and assuming a minute ventilation of 6 l/min (∼8.6 m^3^ over 24 hours) for a healthy adult at rest, the total dose of PM inhaled over 24 hours would be 4,614 μg [19]. Exposure of a human to a daily dose of 4,614 μg of PM would correspond to more than 35 μg of PM exposure for a mouse (25 grams) with minute ventilation of 35–50 ml/min [19]. The dose we tested here (15 µg/mouse every 2^nd^ day) is lower than the comparative human dose of±35 µg/mouse/24 h reported by Mutlu et al. [19]. However, one should take into account that this estimation does not consider particles deposited per surface area of the lung. Also, our study was performed on particles of a nanometer to 1 micrometer diameter [14], [15], whereas the study of Mutlu et al.[19] used PM10. Therefore, the dose of particles given in the present study in terms of reactive surface area will be higher than that of available surface area of PM10 reported previously [19]. Mice were exposed to DEP by i.t. instillation because it provides more accurate dosing, given that mice are nose breathers that filter most inhaled particles [22].

Our data show that repeated exposure to DEP causes a significant inflammatory reaction in the lung characterized by an increase of macrophages and neutrophils in BAL fluid. Similar observations after single dose exposure to particles have been reported in mice hamsters or in rats [19], [23]–[26]. In humans, an increase in the number of neutrophils and mast cell in bronchial submucosa, as well as interleukin-8 and myeloperoxidase concentrations in bronchial lavage have been previously reported [27], [28]. Along with inflammatory cell influx, we found a significant increase in the concentration of TNFα in BAL fluid. This finding corroborates the results of previous studies which have reported an increase of TNFα by alveolar macrophages in particle-exposed mice [25], [29] and humans [30], [31]. Although we did not find an increase of IL-6 in BAL 48 h after the last exposure, this does not necessarily exclude its release at earlier time point of exposure to DEP. Indeed, we have recently shown that exposure to single dose of DEP caused a significant increase of IL-6 at 18 h time point but not at 4 or 24 h post-exposure [8], [32]. Besides causing lung inflammation, repeated exposure to DEP induced an increase in airway resistance assessed by forced oscillation technique after increasing concentration of metacholine. Interestingly, the baseline of airway resistance in DEP exposed mice was higher compared to that in control group. The airway resistance has further dose-dependently increased following increasing dose of metacholine. This effect can be ascribed to the inflammatory reaction taking place in the airways, i.e. influx of inflammatory cells and release of TNFα that caused the observed airway hyperreactivity. We have recently demonstrated that single dose of DEP (30 µg/mouse) caused a significant increase in airway resistance. However, the baseline of airway resistance between control and DEP was not affected. Furthermore, compared to their respective controls, the extent of increase in airway resistance observed in the present study is greater than that observed after exposure to single dose of DEP [8]. A number of studies have found increased risks of asthma outcomes in children and adults who live near roadways with high traffic counts [33]. Evidence of airway inflammation has been observed in healthy volunteers in multiple studies [34], and increased airway hyper-responsiveness has been seen in asthmatic subjects [33].

Although the precise mechanism leading to cardiovascular morbidity and mortality caused by particles is still not fully understood, several studies have shown that systemic inflammation may be a key step in these pathological process through the release of inflammatory mediators [1]. Our data show that repeated exposure to DEP causes systemic inflammation. In fact, we found a significant increase of CRP and TNFα in plasma. An increase of plasma CRP has been recently reported in diabetic mice during inflammation [35], [36]. The absence of increase of plasma IL-6 48 hours after the last exposure to DEP in association with the increase of CRP may appear contradictory given the requirement for IL-6 to induce CRP expression [37]. This, however, does not exclude the release of IL-6 at earlier time point, and suggests that the kinetic of release of these two inflammatory markers is different at least 48 hours after the last exposure to DEP. Additional studies are required to clarify this point. Our findings are in line with human studies that found that risk of cardiovascular events, including myocardial infarction is associated with increased blood levels of inflammatory cytokines such as TNF-α and its receptors, adhesion molecules, and CRP [30], [38], [39].

We have recently demonstrated that single i.t. exposure to DEP (30 µg/mouse) in healthy mice caused a decrease in SBP at 4 and 18 h post exposure [8]. This effect may be due to the dose of particles administered in this study causing cardiac contractile dysfunction [40]. Here, we show that lower doses of DEP (15 µg/mouse) given recurrently every 2^nd^ day for 6 days lead to an increase of SBP which was significant 48 h after the last exposure. A significant increase in SBP was even observed 48 h after the 2^nd^ and 3^rd^ exposure to DEP (data not shown). Most of the studies demonstrated that BP increases only a few days (lags 2 to 5) after an elevation in ambient PM_2.5_ levels and/or following a longer duration (2 to 30 days) of exposure [40]. Our observed increase of SBP could be related to the increase TNFα and CRP leading endothelial and/or smooth muscle dysfunction. Indeed, prolonged PM_2.5_ exposures have proven capable of sensitizing the vasculature to a variety of vasoactive mediators thus tipping vasomotor balance towards vasoconstriction [40]. Our results are in agreement with earlier findings which reported that prolonged exposures to particulate air pollution caused an inflammatory response (release of cytokines) within the systemic circulation. Markers of oxidative stress were also increased [40], [41]. These responses probably played a causal role in the observed impairment in endothelial cell function and vasomotor balance [40], [41]. Further studies have substantiated that PM_2.5_ can induce arterial vascular dysfunction likely via systemic inflammation/oxidative stress-dependent pathways [40], [41].

In the present study, we have assessed the effect of DEP on coagulation events by measuring a set of indices, i.e. thrombosis assessment in pial arterioles *in vivo*, platelet numbers and measurement of circulating PAI-1, soluble vWF and D-dimer. Our data show that repeated exposure to DEP cause thrombotic events in pial arterioles and a decrease of platelet numbers suggestive of platelet aggregation that occurred *in vivo*. A decrease of platelet number after exposure to particles has been reported both in mice [7] and clinical studies [42]. As for SBP, this observed prothrombotic effects can be ascribed the release of TNFα and CRP. A positive correlation of CRP and coronary artery disease, which could be explained by the atherogenic effects of continuing inflammation has been previously described [43]. An association between minor but persistent elevation of serum CRP concentration and future major cardiovascular events has also been shown [44]. Elevated concentrations of proinflammatory cytokines such as TNFα, and CRP play a significant role in the genesis of atherosclerosis and in plaque instability [43]. We found a significant increase of circulating PAI-1 following repeated exposure to DEP. Raised concentrations of circulating PAI-1 have been recognized as an independent risk factor for the development of ischemic cardiovascular events and have been associated with inflammation and atherosclerosis [45], [46]. A recent study reported an increase in PAI-1 mRNA and protein concentrations in lung and adipose tissue of mice treated with concentrated ambient particulate matter or PM [29]. While the concentrations of soluble vWF were not affected by repeated exposure to DEP, plasma concentration of D-dimer, the primary degradation product of cross-linked fibrin, was significantly increased. This effect has, to the best of our knowledge, not been reported before. Plasma concentrations of D-dimer have been found to be significantly raised in several acute thrombotic disorders [47], [48]. There is a growing evidence that there may be an association between elevated concentrations of D-dimer and increased risk of future myocardial infarction [47], [48]. However, only few epidemiological studies have studied the association between particulate air pollution and D-dimer concentrations [39], [49]. The results reported did not show a significant correlation between concentration of PM and those of D-dimer [39], [49]. Additional experimental, clinical and epidemiological studies are needed to clarify this point.

To our knowledge, no study, to date, has addressed the effect of curcumin on the cardiovascular and pulmonary effects following DEP exposure. Because of the observed inflammatory reaction caused by DEP, mice were pretreated with curcumin. The latter is reported to possess a numerous of beneficial activities, such as antitumor, antioxidant, and antiinflammatory activities [9]. It has been shown to interfere with the activities of NF-κB, cytochrome P450, and β amyloid accumulation, production of inflammatory cytokines, and the activity of p300 [18], [50]. Human studies indicate that curcumin is tolerated in large oral doses without apparent toxicity [51]. The dose (45 mg/kg) used in the present study was selected from previously published studies which has been reported to correct cystic fibrosis defect in mice [18] and to inhibit tumour growth in a NCI-H460 xenograft mouse model in vivo [52]. This dose has been reported to correspond, on the basis of mg/kg scaling, to doses of commercially available curcumin products that are routinely consumed [18]. Our data show that pretreatment with curcumin prevented the influx of inflammatory cell in the BAL fluid, and the increase of airway resistance caused by repeated exposure to DEP. Interestingly, the concentration of TNFα in BAL returned to control level suggesting a pivotal role of this proinflammatory cytokine in the observed respiratory levels. Similarly, curcumin pretreatment suppressed the DEP-induced elevation of TNFα and CRP in plasma confirming its potent inflammatory effects. Earlier studies showed that curcumin inhibits TNFα-dependent NFκB activation and blocks the TNF α mediated downregulation of PPAR γ in mesangial cells [53]. Besides inhibiting systemic inflammation, curcumin pretreatment potently prevented the increase of SBP caused by repeated exposure to DEP. Curcumin has been reported to prevent cardiac hypertrophy in salt sensitive Dahl rats by exerting a beneficial preservation of systolic function [54]. The same authors also showed that the acetylation of GATA4 that normally accompanies hypertension was reduced by curcumin [54].

Curcumin pretreatment prevented the increase of PAI-1 and D-dimer concentrations in plasma, both of which play important roles in coronary thrombosis and arteriosclerosis [47], [48], [55]. It has been reported that TNFα is a strong agonist for PAI-1 expression and has been found to play an important role in PAI-1 regulation in a variety of diseases. In a mouse endoxemia model, TNFα has been found to contribute to the lipopolysaccharide-induced PAI-1 expression [56]. In the obesity-associated elevation of PAI-1, evidence also points to TNFα as an important regulator of PAI-1 expression in adipose tissue [55]. Likewise, in line with our results, it has been shown that TNF-alpha, but not IL-6, stimulates PAI-1 expression in human subcutaneous adipose tissue [57], [58]. Our finding corroborates the recent study of Budinger et al. [29] which demonstrated that ambient PM-induced upregulation of PAI-1, disappeared upon treatment of mice with a TNFα inhibitor [29]. This confirms a°pivotal role of TNFα in the observed effects. The effect of curcumin on pial arteriolar thrombosis, a model that depends mainly on platelet activation and aggregation [7], showed a partial but significant inhibition. Likewise, the decrease of platelet numbers caused by repeated exposure to DEP was partially prevented by curcumin. The partial inhibition of curcumin on DEP-induced thrombosis in pial arterioles and decrease in circulating platelet numbers suggest that DEP also exerts a direct effect on platelet aggregation. We and others have previously showed that DEP cause platelet aggregation ex-*vivo* and *in vitro* [6], [8], [59].

In conclusion, this work has shown that repeated exposure to DEP induced airway inflammation and hyperreactivity, systemic inflammation, increased SBP, and accelerated coagulation. TNF α production was increased both in BAL and plasma. Pretreatment with curcumin significantly inhibited the release of TNF α and prevented the respiratory and cardiovascular effects. Further studies using TNFα knockout mice are needed to confirm the central role of TNFα in the observed cardiopulmonary effects. Our findings indicate that curcumin is a potent anti-inflammatory agent that protects against the cardiopulmonary effects of DEP. Our data are in line with previous studies which reported the beneficial anti-inflammatory effect of curcumin on lipopolysaccharides-induced lung inflammation and edema [60], cigarette smoke-induced pulmonary inflammation and emphysema [13] or bleomycin-induced pulmonary fibrosis [12]. Our findings may have therapeutic implications for the potential use of curcumin in prevention of the pulmonary and cardiovascular effects of pollutant particles.

## References

[1] Franchini M, Mannucci PM (2011). Thrombogenicity and cardiovascular effects of ambient air pollution.. Blood.

[2] Brook RD, Rajagopalan S, Pope CA, Brook JR, Bhatnagar A (2010). Particulate Matter Air Pollution and Cardiovascular Disease An Update to the Scientific Statement From the American Heart Association.. Circulation.

[3] Vermylen J, Nemmar A, Nemery B, Hoylaerts MF (2005). Ambient air pollution and acute myocardial infarction.. J Thromb Haemost.

[4] Mills NL, Tornqvist H, Gonzalez MC, Vink E, Robinson SD (2007). Ischemic and thrombotic effects of dilute diesel-exhaust inhalation in men with coronary heart disease.. N Engl J Med.

[5] Mills NL, Donaldson K, Hadoke PW, Boon NA, MacNee W (2009). Adverse cardiovascular effects of air pollution.. Nat Clin Pract Cardiovasc Med.

[6] Nemmar A, Hoet PH, Dinsdale D, Vermylen J, Hoylaerts MF (2003). Diesel exhaust particles in lung acutely enhance experimental peripheral thrombosis.. Circulation.

[7] Nemmar A, Al Salam S, Dhanasekaran S, Sudhadevi M, Ali BH (2009). Pulmonary exposure to diesel exhaust particles promotes cerebral microvessel thrombosis: protective effect of a cysteine prodrug l-2-oxothiazolidine-4-carboxylic acid.. Toxicology.

[8] Nemmar A, Al-Salam S, Zia S, Marzouqi F, Al-Dhaheri A (2011). Contrasting actions of diesel exhaust particles on the pulmonary and cardiovascular systems and the effects of thymoquinone.. Br J Pharmacol.

[9] Ali BH, Marrif H, Noureldayem SA, Bakheit AO, Blunden G (2006). Some biological properties of curcumin: A review.. Natural Product Communications.

[10] Maheshwari RK, Singh AK, Gaddipati J, Srimal RC (2006). Multiple biological activities of curcumin: a short review.. Life Sci.

[11] Shishodia S, Potdar P, Gairola CG, Aggarwal BB (2003). Curcumin (diferuloylmethane) down-regulates cigarette smoke-induced NF-kappaB activation through inhibition of IkappaBalpha kinase in human lung epithelial cells: correlation with suppression of COX-2, MMP-9 and cyclin D1.. Carcinogenesis.

[12] Punithavathi D, Venkatesan N, Babu M (2000). Curcumin inhibition of bleomycin-induced pulmonary fibrosis in rats.. Br J Pharmacol.

[13] Suzuki M, Betsuyaku T, Ito Y, Nagai K, Odajima N (2009). Curcumin attenuates elastase- and cigarette smoke-induced pulmonary emphysema in mice.. Am J Physiol Lung Cell Mol Physiol.

[14] Nemmar A, Al Maskari S, Ali BH, Al Amri IS (2007). Cardiovascular and lung inflammatory effects induced by systemically administered diesel exhaust particles in rats.. Am J Physiol Lung Cell Mol Physiol.

[15] Saber AT, Bornholdt J, Dybdahl M, Sharma AK, Loft S (2005). Tumor necrosis factor is not required for particle-induced genotoxicity and pulmonary inflammation. Arch Toxicol 79: 177–182..

[16] Ying Z, Yue P, Xu X, Zhong M, Sun Q (2009). Air pollution and cardiac remodeling: a role for RhoA/Rho-kinase.. Am J Physiol Heart Circ Physiol.

[17] Vanoirbeek JA, Tarkowski M, Ceuppens JL, Verbeken EK, Nemery B (2004). Respiratory response to toluene diisocyanate depends on prior frequency and concentration of dermal sensitization in mice.. Toxicol Sci.

[18] Egan ME, Pearson M, Weiner SA, Rajendran V, Rubin D (2004). Curcumin, a major constituent of turmeric, corrects cystic fibrosis defects. Science 304: 600–602..

[19] Mutlu GM, Green D, Bellmeyer A, Baker CM, Burgess Z (2007). Ambient particulate matter accelerates coagulation via an IL-6-dependent pathway.. J Clin Invest.

[20] Brook RD, Franklin B, Cascio W, Hong YL, Howard G (2004). Air pollution and cardiovascular disease-A statement for healthcare professionals from the expert panel on population and prevention science of the American Heart Association.. Circulation.

[21] U.N.Environment Program and WHO Report (1994). Air Pollution in the world's megacities. A Report from the U.N. Environment Programme and WHO.. Environment.

[22] Driscoll KE, Costa DL, Hatch G, Henderson R, Oberdorster G (2000). Intratracheal instillation as an exposure technique for the evaluation of respiratory tract toxicity: uses and limitations.. Toxicol Sci.

[23] Nemmar A, Hoet PHM, Vermylen J, Nemery B, Hoylaerts MF (2004). Pharmacological stabilization of mast cells abrogates late thrombotic events induced by diesel exhaust particles in hamsters.. Circulation.

[24] Nemmar A, Melghit K, Ali BH (2008). The Acute Proinflammatory and Prothrombotic Effects of Pulmonary Exposure to Rutile TiO2 Nanorods in Rats.. Exp Biol Med (Maywood ).

[25] Kido T, Tamagawa E, Bai N, Suda K, Yang HH (2010). Particulate Matter Induces IL-6 Translocation from the Lung to the Systemic Circulation.. Am J Respir Cell Mol Biol.

[26] Nemmar A, Al Salam S, Zia S, Yasin J, Al Husseni I (2010). Diesel Exhaust Particles in the Lung Aggravate Experimental Acute Renal Failure.. Toxicological Sciences.

[27] Salvi S, Blomberg A, Rudell B, Kelly F, Sandstrom T (1999). Acute inflammatory responses in the airways and peripheral blood after short-term exposure to diesel exhaust in healthy human volunteers.. Am J Respir Crit Care Med.

[28] Behndig AF, Mudway IS, Brown JL, Stenfors N, Helleday R (2006). Airway antioxidant and inflammatory responses to diesel exhaust exposure in healthy humans.. Eur Respir J.

[29] Budinger GRS, Mckell JL, Urich D, Foiles N, Weiss I (2011). Particulate Matter-Induced Lung Inflammation Increases Systemic Levels of PAI-1 and Activates Coagulation Through Distinct Mechanisms.. Plos One 6.

[30] Delfino RJ, Staimer N, Tjoa T, Gillen DL, Polidori A (2009). Air Pollution Exposures and Circulating Biomarkers of Effect in a Susceptible Population: Clues to Potential Causal Component Mixtures and Mechanisms.. Environ Health Perspect.

[31] Brook RD, Urch B, Dvonch JT, Bard RL, Speck M (2009). Insights into the mechanisms and mediators of the effects of air pollution exposure on blood pressure and vascular function in healthy humans.. Hypertension.

[32] Nemmar A, Al Salam S, Dhanasekaran S, Sudhadevi M, Ali BH (2009). Pulmonary exposure to diesel exhaust particles promotes cerebral microvessel thrombosis: Protective effect of a cysteine prodrug L-2-oxothiazolidine-4-carboxylic acid.. Toxicology.

[33] Nordenhall C, Pourazar J, Ledin MC, Levin JO, Sandstrom T (2001). Diesel exhaust enhances airway responsiveness in asthmatic subjects.. Eur Respir J.

[34] Salam MT, Islam T, Gilliland FD (2008). Recent evidence for adverse effects of residential proximity to traffic sources on asthma. Curr Opin Pulm Med 14: 3–8..

[35] Sallam N, Khazaei M, Laher I (2010). Effect of moderate-intensity exercise on plasma C-reactive protein and aortic endothelial function in type 2 diabetic mice. Mediators Inflamm 2010: 149678..

[36] Park NY, Park SK, Lim Y (2011). Long-term dietary antioxidant cocktail supplementation effectively reduces renal inflammation in diabetic mice. Br J Nutr 106: 1514–1521..

[37] Packard RR, Libby P (2008). Inflammation in atherosclerosis: from vascular biology to biomarker discovery and risk prediction.. Clin Chem.

[38] Pai JK, Pischon T, Ma J, Manson JE, Hankinson SE (2004). Inflammatory markers and the risk of coronary heart disease in men and women. N Engl J Med 351: 2599-2610..

[39] Delfino RJ, Staimer N, Tjoa T, Polidori A, Arhami M (2008). Circulating biomarkers of inflammation, antioxidant activity, and platelet activation are associated with primary combustion aerosols in subjects with coronary artery disease. Environ Health Perspect 116: 898-906..

[40] Brook RD, Rajagopalan S (2009). Particulate matter, air pollution, and blood pressure. J Am Soc Hypertens 3: 332–350..

[41] Sun Q, Hong X, Wold LE (2010). Cardiovascular effects of ambient particulate air pollution exposure.. Circulation.

[42] Ruckerl R, Phipps RP, Schneider A, Frampton M, Cyrys J, Oberdorster G, Wichmann HE, Peters A (2007). Ultrafine particles and platelet activation in patients with coronary heart disease-results from a prospective panel study.. Part Fibre Toxicol.

[43] Libby P, Ridker PM, Hansson GK (2011). Progress and challenges in translating the biology of atherosclerosis. Nature 473: 317-325..

[44] Yeh ET (2004). CRP as a mediator of disease. Circulation 109: II11–II14..

[45] Thogersen AM, Jansson JH, Boman K, Nilsson TK, Weinehall L (1998). High plasminogen activator inhibitor and tissue plasminogen activator levels in plasma precede a first acute myocardial infarction in both men and women: evidence for the fibrinolytic system as an independent primary risk factor.. Circulation.

[46] Cesari M, Pahor M, Incalzi RA (2010). Plasminogen activator inhibitor-1 (PAI-1): a key factor linking fibrinolysis and age-related subclinical and clinical conditions. Cardiovasc Ther 28: e72–e91..

[47] Ridker PM, Hennekens CH, Cerskus A, Stampfer MJ (1994). Plasma concentration of cross-linked fibrin degradation product (D-dimer) and the risk of future myocardial infarction among apparently healthy men.. Circulation.

[48] Lowe GD, Rumley A (1999). Use of fibrinogen and fibrin D-dimer in prediction of arterial thrombotic events. Thromb Haemost 82: 667-672.. 99080667 [pii].

[49] Ruckerl R, Ibald-Mulli A, Koenig W, Schneider A, Woelke G (2006). Air pollution and markers of inflammation and coagulation in patients with coronary heart disease.. Am J Respir Crit Care Med.

[50] Epstein JA (2008). Currying favor for the heart. J Clin Invest 118: 850–852..

[51] Cheng AL, Hsu CH, Lin JK, Hsu MM, Ho YF (2001). Phase I clinical trial of curcumin, a chemopreventive agent, in patients with high-risk or pre-malignant lesions.. Anticancer Res.

[52] Su CC, Yang JS, Lu CC, Chiang JH, Wu CL (2010). Curcumin inhibits human lung large cell carcinoma cancer tumour growth in a murine xenograft model. Phytother Res 24: 189–192..

[53] Ghosh SS, Massey HD, Krieg R, Fazelbhoy ZA, Ghosh S (2009). Curcumin ameliorates renal failure in 5/6 nephrectomized rats: role of inflammation. Am J Physiol Renal Physiol 296: F1146-F1157..

[54] Morimoto T, Sunagawa Y, Kawamura T, Takaya T, Wada H (2008). The dietary compound curcumin inhibits p300 histone acetyltransferase activity and prevents heart failure in rats. J Clin Invest 118: 868-878..

[55] Gruber F, Hufnagl P, Hofer-Warbinek R, Schmid JA, Breuss JM (2003). Direct binding of Nur77/NAK-1 to the plasminogen activator inhibitor 1 (PAI-1) promoter regulates TNF alpha-induced PAI-1 expression. Blood 101: 3042–3048..

[56] Yamashita M, Yamashita M (1997). Tumor necrosis factor alpha is involved in the induction of plasminogen activator inhibitor-1 by endotoxin. Thromb Res 87: 165–170.. S0049-3848(97)00116-3 [pii].

[57] Hou B, Eren M, Painter CA, Covington JW, Dixon JD (2004). Tumor necrosis factor alpha activates the human plasminogen activator inhibitor-1 gene through a distal nuclear factor kappaB site. J Biol Chem 279: 18127–18136..

[58] Plomgaard P, Keller P, Keller C, Pedersen BK (2005). TNF-alpha, but not IL-6, stimulates plasminogen activator inhibitor-1 expression in human subcutaneous adipose tissue. J Appl Physiol 98: 2019–2023..

[59] Nemmar A, Al Salam S, Zia S, Dhanasekaran S, Shudadevi M (2010). Time-course effects of systemically administered diesel exhaust particles in rats. Toxicol Lett..

[60] Suresh MV, Wagner MC, Rosania GR, Stringer KA, Min KA (2012). Pulmonary Administration of Water-soluble Curcumin Complex Reduces ALI Severity. Am J Respir Cell Mol Biol..

